# RNA metabolism in neurodegenerative disease

**DOI:** 10.1242/dmm.028613

**Published:** 2017-05-01

**Authors:** Elaine Y. Liu, Christopher P. Cali, Edward B. Lee

**Affiliations:** Translational Neuropathology Research Laboratories, Perelman School of Med. Univ. of Pennsylvania, 613A Stellar Chance Laboratories, Philadelphia, PA 19104, USA

**Keywords:** Disease, RNA binding proteins, Microsatellite repeats, miRNA, tRNA, lncRNA, RNA

## Abstract

Aging-related neurodegenerative diseases are progressive and fatal neurological diseases that are characterized by irreversible neuron loss and gliosis. With a growing population of aging individuals, there is a pressing need to better understand the basic biology underlying these diseases. Although diverse disease mechanisms have been implicated in neurodegeneration, a common theme of altered RNA processing has emerged as a unifying contributing factor to neurodegenerative disease. RNA processing includes a series of distinct processes, including RNA splicing, transport and stability, as well as the biogenesis of non-coding RNAs. Here, we highlight how some of these mechanisms are altered in neurodegenerative disease, including the mislocalization of RNA-binding proteins and their sequestration induced by microsatellite repeats, microRNA biogenesis alterations and defective tRNA biogenesis, as well as changes to long-intergenic non-coding RNAs. We also highlight potential therapeutic interventions for each of these mechanisms.

## Abstract (precis)

**Summary:** In this At a Glance review, Edward Lee and co-authors provide an overview of RNA metabolism defects, including mislocalization of RNA-binding proteins and microRNA biogenesis alterations, that contribute to neurodegenerative disease pathology.

## Introduction

Aging-related neurodegenerative diseases, such as Alzheimer's disease (AD), Parkinson's disease (PD), frontotemporal degeneration (FTD) and amyotrophic lateral sclerosis (ALS), among others, are relentlessly progressive and uniformly fatal neurological diseases that are characterized by irreversible neuron loss and gliosis. Although dementia prevalence as a percentage of the elderly has declined in developed countries, the absolute number of dementia cases is growing as a result of an increase in the aging population (Langa et al., 2017). Thus, it is important for us to better understand the basic biological mechanisms that contribute to neurodegeneration.

**Figure:**
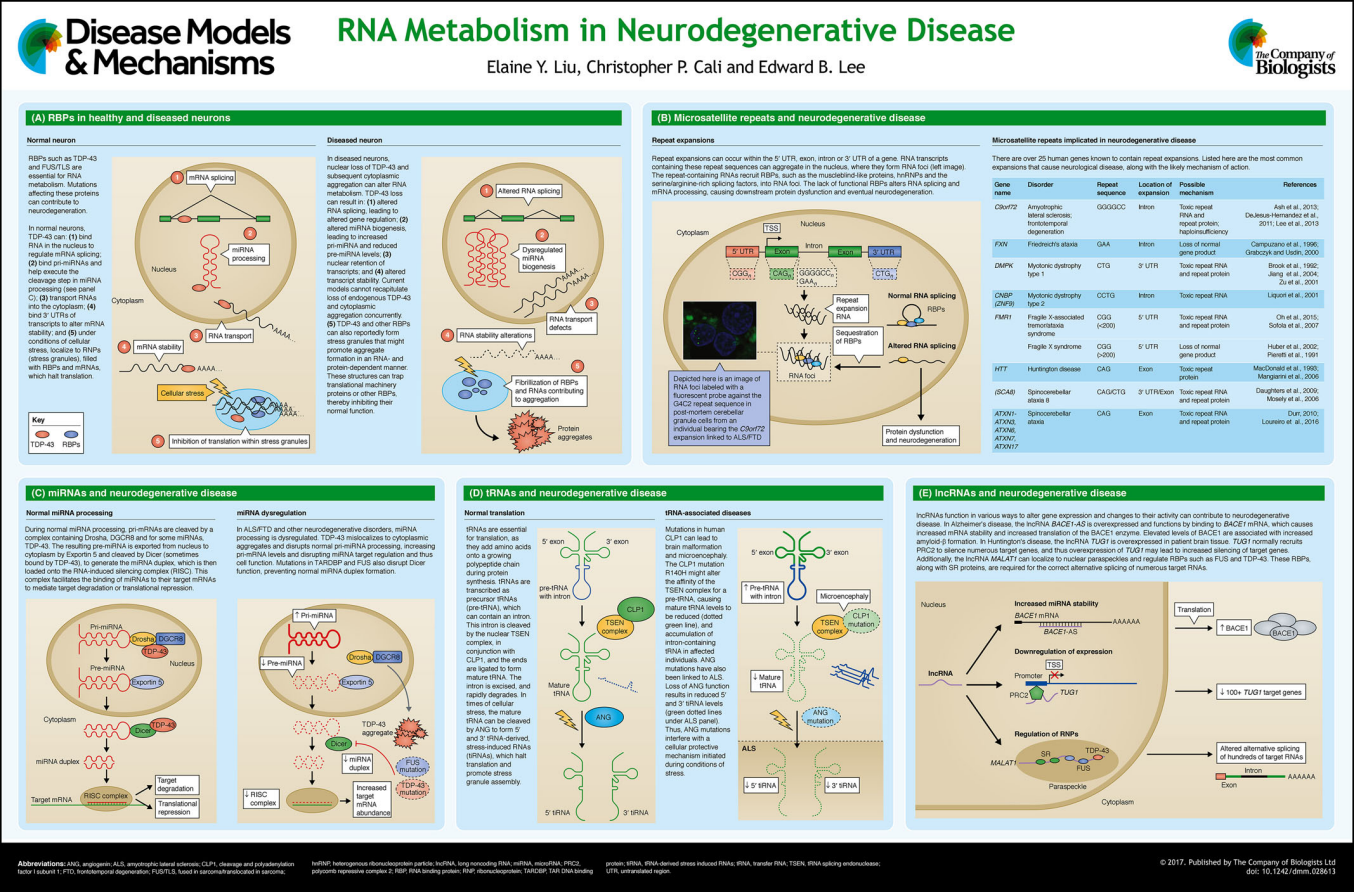
A high-resolution version of the poster is available for downloading at http://dmm.biologists.org/lookup/doi/10.1242/dmm.028613.supplemental.

Although much emphasis has been placed on the role of protein aggregates in neurodegenerative diseases, multiple lines of evidence also converge on altered RNA processing as a contributing factor in the pathogenesis of these diseases (Anderson and Ivanov, 2014; Belzil et al., 2013; Bentmann et al., 2013; Halliday et al., 2012; Ling et al., 2013). Defects at all levels of gene regulation, from RNA synthesis, processing, function and degradation, are associated with disease-specific alterations in RNA-binding proteins (RBPs), and in non-coding RNAs, such as microRNAs (miRNA), transfer RNAs (tRNA) and long-noncoding RNAs (lncRNA). Given that these basic processes are essential for normal and properly regulated gene expression, it is increasingly clear that aberrations in these processes can contribute to disease. In this Review and accompanying poster, we highlight several key themes that explain how different classes of RNAs or RBPs can impair gene regulation. We also highlight specific examples with evidence to show that improper RNA metabolism is a critical feature of neurodegeneration.

## RNA-binding proteins regulate RNA metabolism

RBPs are essentially required at all levels of RNA processing in both the nucleus and cytoplasm where transcription, splicing, RNA stabilization, and RNA degradation occur (see poster panel A). Two notable examples of RBP defects occur in familial and sporadic cases of ALS and FTD. ALS is a neurodegenerative disease that leads to the loss of upper and lower motor neurons from the motor cortex and spinal cord, respectively, whereas FTD is associated with neuronal loss in the temporal and frontal cortex. Despite different areas of neuronal atrophy, a common link between ALS and FTD patients is a nuclear RBP called TAR DNA-binding protein-43 (TDP-43). Post-mortem brains from human ALS and FTD patients show a characteristic mislocalization of TDP-43 from the nucleus into phosphorylated, ubiquitylated cytoplasmic TDP-43 aggregates (see poster panel A) (Neumann et al., 2006). Indeed, rare disease-causing mutations in *TARDBP*, the gene encoding TDP-43, suggest that TDP-43 dysfunction is sufficient to cause ALS (Van Deerlin et al., 2008; Gitcho et al., 2008; Kabashi et al., 2008; Sreedharan et al., 2008), although the mechanism by which these mutations cause disease is unclear. TDP-43 functions ubiquitously in RNA processing, including splicing (Buratti et al., 2001; Ling et al., 2015; Shiga et al., 2012; Tollervey et al., 2011), stability (Costessi et al., 2014; Liu et al., 2012; Strong et al., 2007) and transport (Alami et al., 2014). Shortly after the discovery of *TARDBP* mutations, mutations in *FUS*, which encodes the nuclear protein fused in sarcoma (FUS) (also known as translated in liposarcoma, TLS), were identified in a subset of individuals with ALS, and FUS was revealed to be mislocalized to the cytoplasm (Kwiatkowski et al., 2009; Vance et al., 2009). Similar to TDP-43, FUS interacts with serine arginine (SR) proteins involved in RNA splicing (Yang et al. 1998) and regulates transcription by recruiting RBPs through non-coding RNAs (Wang et al., 2008). An additional example of abnormally localized nuclear RBPs is evident in individuals with multisystem proteinopathy (MSP), in whom mutations in the gene encoding heterogeneous nucleoriboprotein particle A1 (*HNRNPA1*) and A2B1 (*HNRNPA2B1*) contribute to disease. MSP is characterized by the progressive degeneration of muscle, brain, motor neurons and bone, which sometimes manifests as ALS or FTD (Kim et al., 2013). Mutations in the gene encoding valosin-containing protein (*VCP*), a triple ATPase protein involved in many cellular functions including endolysosomal degradation, autophagy, and the ubiquitin proteasome system, also causes MSP (Watts et al., 2004).

It is thought that the loss of RBPs through their nuclear mislocalization and/or the toxicity caused by their cytoplasmic aggregation can lead to disease, but their relative contributions to disease remain unclear. Mouse models where antisense oligonucleotides (ASOs) against *Tardbp* depleted TDP-43, show altered global RNA expression affecting 601 genes, and specifically neuronal genes with long introns (Polymenidou et al., 2011). To better understand the targets of these RBPs, TDP-43 and FUS have been immunoprecipitated from mice brains and rat primary neuronal cultures, revealing that these proteins bind to non-coding RNA sites, namely introns and 3′ untranslated regions (UTRs) of thousands of genes (Lagier-Tourenne et al., 2012; Sephton et al., 2011). Some of these genes include ncRNAs, like metastasis-associated lung adenocarcinoma transcript 1 (*Malat1*) and nuclear paraspeckle assembly transcript 1 (*Neat1*) (Lagier-Tourenne et al., 2012; Polymenidou et al., 2011; Tollervey et al., 2011). Thus, it is possible that the loss of RBPs influences the processing of these non-coding RNAs and contributes to global RNA dysregulation (see poster panel A). Given that the depletion of these RBPs alters the expression of thousands of genes, it is likely that some, or even all of these changes contribute to disease pathogenesis.

The formation of cytoplasmic RNA granules that lead to cytoplasmic aggregates has also been proposed to be pathogenic. When cells are stressed, cytoplasmic RNA granules that contain stalled translational complexes are formed. TDP-43, FUS, and other RBPs, such as hnRNPA1 and A2B1, localize to stress granules (Kim et al., 2013; Li et al., 2013). Indeed, RBPs with low complexity domains (LCD), such as TDP-43, FUS and hnRNPA1, can phase separate to create dynamic membrane-less organelles or liquid droplets that underlie the transient nature of stress granules (Courchaine et al., 2016; Molliex et al., 2015). The liquid properties of these organelles are dependent on their constituents. Namely, the intrinsic properties, type, concentration of the RBP, RNAs that the RBPs are bound to and the concentration of the RNA greatly influence these RNA granules (Kroschwald et al., 2015; Smith et al., 2016). For example, increasing the concentration of RBPs can reduce the liquid-like properties of these RNA granules, thereby promoting the formation of hydrogels and eventually an insoluble amyloid-like aggregate (Guo and Shorter, 2015; Kato et al., 2012; Lin et al., 2015; Molliex et al., 2015; Xiang et al., 2015). Indeed, disease-associated mutations within the LCDs of RBPs can enhance prion-like properties, and accelerate the shift from liquid to solid and disrupt ribonucleoprotein (RNP) granule formation (Murakami et al., 2015). At present, there is little evidence supporting amyloid-like fibrillar aggregates within neurons in ALS/FTD, but the relationship between other biophysical assemblies such as hydrogels, and neuronal aggregates is being investigated (Murakami et al., 2015). Thus, the formation of liquid droplets is another mechanism by which RBP disruption could contribute to disease (see poster).

The formation of cytoplasmic aggregates has also been implicated in neurodegenerative disease. Several different rat and mouse models, in which wild-type or mutant *TARDBP* or mutant *FUS* bearing ALS-associated mutations are overexpressed, develop cytoplasmic aggregation and exhibit features of ALS and FTD, including cortical and hippocampal neuronal loss and motor deficits (Huang et al., 2011; Igaz et al., 2011; Scekic-Zahirovic et al., 2016; Sharma et al., 2016; Tsai et al., 2010; Wils et al., 2010; Xu et al., 2010). However, none of these models has recapitulated the loss of endogenous nuclear TDP-43 or FUS. Despite the discrepancies between animal models and human pathology, there is *in vitro* evidence in support of the toxicity of cytoplasmic aggregates. Live tracking of rat primary cortical neurons to assess their survival shows that neurons with cytoplasmic TDP-43 have a greater risk of death, and that this risk depends on the amount of cytoplasmic TDP-43 present (Barmada et al., 2010). This corroborates the finding that overexpression of FUS or TDP-43 in yeast results in cytoplasmic aggregation of these proteins (Johnson et al., 2008, 2009; Sun et al., 2011). TDP-43 toxicity is dependent not only on its RNA-binding ability but also its C- terminus (Elden et al., 2010; Johnson et al., 2009; Voigt et al., 2010), the region where most disease-causing *TARDBP* mutations are found (Gitcho et al., 2008; Kabashi et al., 2008; Sreedharan et al., 2008). Although there is no consensus on which mechanism is more toxic, it is likely that both nuclear clearance and cytoplasmic aggregation of RBPs contribute to disease. Indeed, an effort to parse the effects of nuclear TDP-43 loss and TDP-43 cytoplasmic aggregation in a mouse motor neuron-like hybrid cell line (NSC34) shows that both contribute relatively equally to cellular toxicity (Cascella et al., 2016).

At present, potential therapeutic interventions are based on reducing the formation of toxic cytoplasmic aggregates. This is achieved in several ways, for example by: (1) activating the heat shock response; (2) using heat shock protein (Hsp)104 ‘disaggregases’; or (3) by modulating the ubiquitin proteasome system and autophagy. HSPs function as molecular chaperones and are involved in protein folding, protein trafficking and in coping with denatured proteins (Lindquist and Craig, 1988). Prior work has shown that the overexpression of an HSP, heat shock factor 1 (HSF1), in rat primary neuronal cultures overexpressing wild-type TDP-43 prevents cytoplasmic aggregation of TDP-43 by interacting with other HSPs to enhance refolding. This reduces toxicity in a human bone marrow neuroblast cell line (SH-SY5Y) overexpressing either wild-type or mutant TDP-43 (Chen et al., 2016). Components of the heat shock response have also been engineered to remove aggregated proteins. Modified Hsp104 improves its disaggregation capabilities relative to the wild-type Hsp104, and is able to suppress FUS and TDP-43 toxicity in yeast. This provides a potential intervention to eliminate protein aggregates that contribute to toxicity (Jackrel et al., 2014). Enhancing components of the ubiquitin proteasome system or autophagy can also reduce these aggregates. For example, increasing cAMP levels with forskolin in human embryonic kidney cells (293A) enhances the ubiquitin proteasome system to clear aggregation-prone proteins, such as FUS and TDP-43, in cells overexpressing either wild-type or mutant forms of both proteins (Lokireddy et al., 2015). Furthermore, two different studies show that using autophagy activators rescues motor dysfunction in transgenic FTD mice and also improves survival of neurons and astrocytes derived from human induced pluripotent stem cells from ALS patients with a *TARDBP* mutation (Barmada et al., 2014; Wang et al., 2012b). Until the toxic mechanism that underlies RBP pathology is uncovered, it is difficult to determine which therapeutic intervention will be the most beneficial to patients.

## RBP sequestration by microsatellite repeat expansions

Microsatellite repeat expansion disorders are a class of neurodegenerative diseases caused by repetitive DNA elements that form long expansions within gene bodies or in untranslated regions. Over 25 human genes that contain repeat expansions have been identified to date (Loureiro et al., 2016). Some neurodegenerative diseases caused by microsatellite repeat expansion have been linked to the sequestration of RBPs by expanded repeat sequences; these expanded sequences sequester RBPs away from their target RNAs, thereby altering RNA splicing and metabolism (see poster panel B) (Iwahashi et al., 2006; Jiang et al., 2004; Lee et al., 2013).

One example of altered RNA metabolism in neurological disease comes from the expansion of a CTG triplet in the 3′ UTR of the gene *DMPK*, which leads to myotonic dystrophy (DM) (Brook et al., 1992). This expansion is transcribed into repeat RNA that forms aggregates, called RNA foci. These aggregates form within the nuclei of human-derived DM cells (Davis et al., 1997; Taneja et al., 1995) and recruit a class of RBPs that regulate alternative splicing, called the muscleblind-like proteins (MBNLs) (Miller et al., 2000). By sequestering MBNLs into RNA foci, mutant *DMPK* renders MBNLs unable to regulate splicing and the polyadenylation of hundreds of target genes (Batra et al., 2014; Goodwin et al., 2015; Wang et al., 2012a).

Similarly, repeat expansions associated with ALS/FTD and with Fragile X-associated tremor/ataxia syndrome (FXTAS) also sequester RBPs. In FXTAS, a short repeat expansion (<200 repeats) in the untranslated region of the gene *FMR1* is transcribed into RNA and interacts with hnRNPs, MBNL1 and other RBPs (Iwajasjo et al., 2006; Jin et al., 2007; Sofola et al., 2007), thereby altering splicing and microRNA biogenesis in affected individuals (Sellier et al., 2010, 2013).

In the most common inherited form of ALS/FTD, a hexanucleotide (G_4_C_2_) expansion in the first intron of the gene *C9orf72* is bidirectionally transcribed into mutant RNA that forms aggregates in the nucleus (DeJesus-Hernandez et al., 2011; Gendron et al., 2013; Renton et al., 2011; Zu et al., 2013). Current evidence shows that RBPs involved in splicing, such as hnRNPs and the SR splicing factors that comprise the spliceosome, are sequestered by mutant repeat-containing RNA (Cooper-Knock et al., 2014; Lee et al., 2013) (see poster panel B). Additionally, the repeat expansion, which normally regulates vesicle trafficking and autophagy (Yang et al 2016; Aoki et al 2017). can interfere with transcription of the *C9orf72* gene, resulting in haploinsufficiency of the protein product (Burberry et al., 2016; Ciura et al., 2013; DeJesus-Hernandez et al., 2011). Reduced transcription is due, in part, to hypermethylation of the mutant *C9orf72* promoter. Hypermethylation is observed in about one third of *C9orf72* mutation carriers and is associated with reduced mutant RNA accumulation and an attenuated clinical phenotype, suggesting that reduced transcription of mutant *C9orf72* is actually protective against disease (Liu et al., 2014; McMillan et al., 2015; Russ et al., 2015). Thus, altered RNA metabolism is clearly implicated in neurodegenerative diseases caused by repeat expansions.

Given that repeat expansions cause widespread disruption to RNA metabolism, it will be challenging to target downstream processes for therapeutic intervention. Therefore, the most promising therapeutic approaches are those that work upstream to reduce the amount of repeat-containing transcripts. Several studies have used ASOs to target *C9orf72* and DM expansions for degradation via an RNAse H-mediated pathway (Donnelly et al., 2013; Jiang et al., 2016; Lagier-Tourenne et al., 2013; Sareen et al., 2013; Wheeler et al., 2012). In fact, ASOs are already in clinical trial for the treatment of Huntington's disease (HD) (Kordasiewicz et al., 2012) and have been approved for the treatment of spinal muscular atrophy (SMA) (FDA, 2016, http://www.fda.gov/NewsEvents/Newsroom/PressAnnouncements/ucm534611.htm). An alternative to targeting the repeat-containing transcript for degradation is to target proteins that are responsible for transcribing the repeat expansion. For instance, knockdown of the transcription elongation factor *SUPT4H1* selectively decreases the repeat-containing RNA in *C9orf72* expansion fibroblasts derived from human carriers (Kramer et al., 2016). This strategy is attractive because transcription of the repeat is blocked in both the sense and antisense directions (Jiang and Cleveland, 2016; Kramer et al., 2016). A final therapeutic approach involves small molecules that target the expanded RNA to prevent their interactions with RBPs. Several of these compounds have been identified but are still in the early stages of development (Disney et al., 2012; Luu et al., 2016; Su et al., 2014). Although the repeat-expansion disorders offer a clear therapeutic target, reversing alterations in RNA metabolism will be challenging in neurodegenerative diseases that lack a clear genetic etiology.

## MicroRNA dysregulation in neurodegeneration

Genes can also be regulated post-transcriptionally via miRNAs, a class of small non-coding RNAs (18-25 nucleotides). miRNAs are initially transcribed by RNA polymerase II and then undergo sequential cleavage, first by Drosha and DGCR8 in the nucleus – to generate pre-miRNAs from pri-miRNAs – and then by Dicer after being exported into the cytoplasm, to generate a miRNA duplex (Lee et al., 2003). The miRNA duplex is unwound and one of the strands is incorporated into the RNA-induced silencing complex (RISC), where it binds to Argonaute (Agos) proteins (Schwarz et al., 2003). Agos then cleave the mRNA complementary to the miRNA or inhibit cap-dependent mRNA translation, both of which lead to translational repression (Chendrimada et al., 2005; Kiriakidou et al., 2007).

There are two ways in which miRNA dysregulation contributes to neurodegeneration: alterations to miRNA biogenesis or to miRNA expression, both of which can affect disease-associated genes. A notable example of altered miRNA processing is evident in ALS/FTD through TDP-43 function. In normal neurons, TDP-43 binds to Drosha in the nucleus to cleave select pri-miRNAs and to Dicer in the cytoplasm to cleave some pre-miRNAs (Ling et al., 2010). In mouse neuroblastoma cells (Neuro2a), TDP-43 regulates neuronal outgrowth by modulating pri-miRNA-132 production (Kawahara and Mieda-Sato, 2012). Using lysates from NSC-34 motor neuron cells overexpressing ALS-causing mutations in *TARDBP* and *FUS* in a cell-free dicing activity assay, Dicer function was shown to be altered resulting in an inhibition of miRNA biogenesis (Emde et al., 2015) (see poster panel C). Conversely, activation of Dicer with enoxacin in an ALS mouse model that carries a mutation in the gene Cu/Zn superoxide dismustase 1 (*SOD1*) reverses miRNA downregulation and neuromuscular defects (Emde et al., 2015). This finding indicates that impairment of Dicer activity and miRNA downregulation likely contributes to ALS pathogenesis. Similarly, extensive miRNA dysregulation is seen in FXTAS patients, where FXTAS-associated CGG repeats sequester DGCR8 and prevent proper miRNA processing. In primary mouse cortical neuron cultures that express a plasmid containing toxic 60 CGG repeats, overexpression of DGCR8 is sufficient to reverse cellular toxicity (Sellier et al., 2013). These examples demonstrate that improper miRNA processing leads to global miRNA dysregulation and contributes to different neurodegenerative diseases.

In addition to altered miRNA processing, specific miRNAs that affect certain disease-linked genes are also associated with neurodegenerative diseases (see poster). There are notable examples in Alzheimer's disease (AD) and ALS. AD is the most common form of dementia and is characterized by progressive memory loss, impaired cognitive function, and the inability to perform daily tasks. Pathologically, AD is defined by the presence of extracellular amyloid-β (Aβ) plaques and of intracellular hyper-phosphorylated neurofibrillary tangles composed of tau (Hardy and Selkoe, 2002). Beta-site APP-cleaving enzyme 1 (BACE1) and γ-secretase cleave amyloid precursor protein (APP), resulting in Aβ peptides that accumulate into plaques (Vassar et al., 1999). Multiple miRNAs have been implicated in Aβ production via BACE1 modulation and in tau phosphorylation that leads to hyperphosphorylated neurofibrillary tangle formation. Additionally, multiple miRNAs have also been implicated in ALS pathogenesis or as biomarkers of disease. For example, miR-23a is overexpressed in skeletal muscle biopsies from ALS patients. This miR-23a has been shown to regulate peroxisome proliferator-activated receptor γ coactivator-1α, a regulator of mitochondrial biogenesis and function (Russell et al., 2013), which is important for skeletal muscle function. Using spinal cord from individuals with ALS, miR-155-5p and miR-142-5p are upregulated whereas let-7e, miR-148-5p, miR-133b, miR-140-3p and miR-577 are downregulated. These miRNAs regulate neuronal homeostasis, pathogenesis of ALS and other neurodegeneration-related transcripts ranging from ubiquilin, RNA-binding protein fox-1 and reelin among others (Figueroa-Romero et al., 2016) (see Table 1). Indeed, miRNA dysregulation can affect a range of disease-associated targets, which can contribute to neurodegeneration.

**Table 1.**
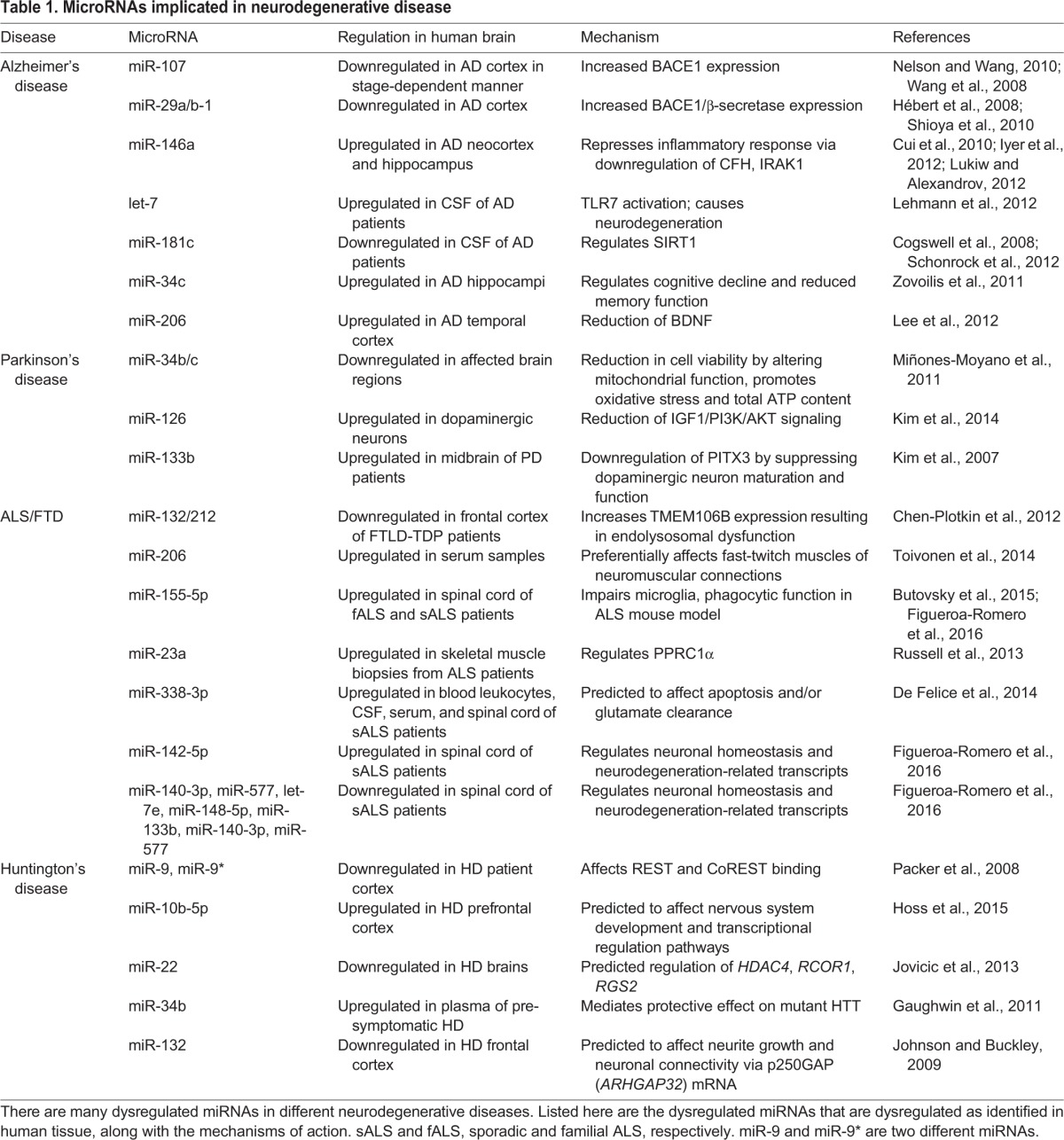
**MicroRNAs implicated in neurodegenerative disease**

Altered miRNA signatures can also indicate potential diagnostic biomarkers. Indeed, various miRNA studies have identified differentially expressed miRNAs in post-mortem tissue or blood and in cerebrospinal fluid (CSF) that differ by disease stage (see Table 1) (Cogswell et al., 2008; Lau et al., 2013; Wang et al., 2011). Furthermore, miRNA-based therapeutics, such as miRNA mimics or miRNA antagonists (antagomirs), have been designed to either reverse the downregulation or upregulation of disease-associated miRNAs, respectively. These have been investigated for the treatment of cancer and cardiovascular disease (Broderick and Zamore, 2011; Thum, 2012; Wu et al., 2007b) but few have been used to treat neurodegeneration. One example of an antagomir in neurodegeneration comes from an AD mouse model (Tg2576) characterized by elevated levels of Aβ and the presence of amyloid plaques. The treatment of Tg2576 with an antagomir against miR-206, which targets brain-derived neurotrophic factor (*Bdnf*), increases BDNF levels and improves memory function (Lee et al., 2012). Another example is based on the finding that miR-155 is increased in spinal cord from ALS patients and in an ALS mouse model with a mutation in *SOD1.* A locked nucleic acid (modified RNA nucleotide) anti-miR-155 reduces miR-155 levels in this mouse (Butovsky et al., 2015; Figueroa-Romero et al., 2016), thereby increasing survival and restoring the abnormal microglia and monocyte inflammatory signature (Butovsky et al., 2015). Because of the burgeoning importance of miRNAs in disease, it seems important to first investigate and develop an miRNA signature that is validated as a biomarker of disease by independent studies. Then, therapeutic interventions can be designed to target these specific miRNAs.

## Other non-coding RNAs in neurodegeneration

With the discovery that other classes of non-coding (nc)RNAs are important for gene expression (Cech and Steitz, 2014), it is perhaps unsurprising that alterations to some of these other ncRNAs, such as to tRNAs and lncRNAs, can also lead to neurodegeneration. tRNAs are essential for translation; they use aminoacyl-tRNA synthetase to attach amino acids to the tRNA molecule, which then transfer the appropriate amino acid to a growing polypeptide chain during protein synthesis. tRNA biogenesis entails the transcription and splicing of tRNAs by complexes that involve the proteins CLP1 (cleavage and polyadenylation factor I subunit 1) and TSEN (the tRNA-splicing endonuclease complex) (Paushkin et al., 2004; Trotta et al., 2006) (see poster panel D). Additionally, during cellular stress, angiogenin (ANG) cleaves tRNAs into fragments that might inhibit translation or target specific mRNAs for degradation as a cellular protective mechanism (Fu et al., 2009; Ivanov et al., 2011; Yamasaki et al., 2009). Indeed, mutations in tRNA biogenesis components lead to neurodegeneration. Mutations in aminoacyl-tRNA synthetases are found in individuals with Charcot–Marie–Tooth, a disease of peripheral neuropathy (Antonellis et al., 2003; Nangle et al., 2007; Xie et al., 2007). It is unclear why abnormalities in tRNA biogenesis result in different peripheral neuropathies but various mechanisms have been proposed, including: (1) loss of function of tRNA loading and subsequent protein translation inhibition; (2) a dominant-negative effect whereby mutant protein interferes with the wild-type protein activity; and (3) impaired axonal transport due to tRNA synthetase mutations, among others (Motley et al., 2010; Niehues et al., 2015). A mutation in *CLP1* (R140H) is a cause of progressive brain atrophy and microcephaly in humans (Karaca et al., 2014; Schaffer et al., 2014). *CLP1* R140H renders CLP1 unable to interact with TSEN, resulting in altered tRNA processing (see poster panel D). Mutations in *ANG* that might contribute to the loss of ANG function also occur in ALS (Greenway et al., 2004, 2006; Wu et al., 2007b). Altered translation can also contribute to neurodegenerative disease. Specifically, a mutation in *GTPBP2*, which encodes a ribosome rescue factor, leads to global ribosomal stalling by epistatically interacting with an isodecoder of nuclear-encoded tRNAs, called n-Tra20. This aberrant interaction ultimately leads to neurodegeneration via altered translation (Ishimura et al., 2014). These studies only provide a snapshot of how aberrations in tRNA processing can lead to neurodegeneration.

lncRNAs have also been implicated in neurodegenerative diseases (see poster panel E). These RNAs are longer than 200 nucleotides and fulfill various functions, including acting as scaffolds for chromatin modifiers and nuclear paraspeckles, as transcriptional co-regulators, and even as decoys for other RNAs (Prensner and Chinnaiyan, 2011). Alterations in lncRNAs can affect any one of these processes, thereby contributing to neurodegeneration. lncRNAs associated with disease can post-transcriptionally increase gene expression, as seen with the lncRNAs, *BACE1*-antisense (AS) and lnc-SCA7 (official symbol ATXN7L3B). BACE1-AS is increased in AD brains and competes with miRNA-545-5p binding to stabilize *BACE1* mRNA. This is associated with increased *BACE1* expression and with the formation of the Aβ peptides that contribute to AD pathology (see poster panel E) (Faghihi et al., 2008). In spinocerebellar ataxia type 7 (SCA7), mutant CAG repeats in the *ATXN7* gene contribute to cerebellar neuronal death. Normally, ATXN7L3B regulates ATXN7, which is loaded into a transcriptional activator complex, called STAGA. STAGA promotes miR-124 biogenesis, which in turn, represses ATXN7L3B expression. In SCA7, mutant CAG repeats promote mutant ATXN7 protein levels, which reduce STAGA activity. This consequently reduces miR-124 biogenesis, increases ATXN7L3B expression and promotes more mutant ATXN7 production (Tan et al., 2014). Other examples of genes with altered lncRNAs include *MALAT1* and *NEAT1*, which are important for splicing and synapse formation (Bernard et al., 2010; Tripathi et al., 2010)*.* Both of these RNAs are bound by TDP-43 and FUS (Lagier-Tourenne et al., 2012; Polymenidou et al., 2011; Tollervey et al., 2011). *MALAT1* and *NEAT1* have been shown to colocalize in nuclear paraspeckles, sites where RNA is retained to control gene expression during different cell processes (see poster panel E) (Fox and Lamond, 2010). Additionally, *NEAT1* is upregulated in the HD brain, which is thought to make cells susceptible to oxidative stress (Johnson, 2012; Sunwoo et al., 2016). Other HD-associated genes with lncRNAs include *TUG1*, which is increased in HD and which normally associates with polycomb repressive complex 2 (PRC2) to repress gene expression (Johnson, 2012; Khalil et al., 2009) (see poster). Thus, lncRNAs are likely to influence gene expression post-transcriptionally to contribute to neurodegenerative disease.

Therapies designed to target lncRNAs involve inhibiting the function of lncRNAs usually by: (1) blocking the interaction of the antisense and sense mRNA by degrading the antisense strand, which leads to the transcriptional repression of the gene; (2) using aptamers to bind and inhibit lncRNA structures and prevent their activity; and (3) employing small molecules that inhibit lncRNA interactions (Fatemi et al., 2014; Sullenger and Nair, 2016). Although it has been shown that treating a mutant APP-expressing human HEK-SW cell line with siRNA against *BACE1*-AS leads to reduced Aβ (Faghihi et al., 2008), this finding has not been validated in an AD mouse model or human patients. A better understanding of how specific lncRNAs contribute to the disease phenotype is integral to designing better-targeted therapeutics for these diseases.

## Conclusions

Neurodegenerative diseases occur by different means and present with various pathologies. However, it is becoming increasingly clear that altered or defective RNA metabolism, including mislocalized RBPs and aberrant ncRNA biogenesis or expression, can contribute to neurodegeneration. Because numerous disease-associated pathways are perturbed in these neurodegenerative diseases (as summarized in the accompanying poster), it is unlikely that targeting only one of these modalities will lead to a complete cure. Nonetheless, reversing some of these RNA aberrations could prove to be effective in modifying the incessantly downward disease trajectory associated with these diseases. Perhaps by modifying disease progression, such approaches could provide a significant benefit for those afflicted by neurodegenerative disease.

## References

[DMM028613C1] Alami N. H., Smith R. B., Carrasco M. A., Williams L. A., Winborn C. S., Han S. S. W., Kiskinis E., Winborn B., Freibaum B. D., Kanagaraj A. et al. (2014). Axonal transport of TDP-43 mRNA granules is impaired by ALS-causing mutations. *Neuron* 81, 536-543. 10.1016/j.neuron.2013.12.018 24507191 PMC3939050

[DMM028613C2] Anderson P. and Ivanov P. (2014). tRNA fragments in human health and disease. *FEBS Lett.* 588, 4297-4304. 10.1016/j.febslet.2014.09.001 25220675 PMC4339185

[DMM028613C3] Antonellis A., Ellsworth R. E., Sambuughin N., Puls I., Abel A., Lee-Lin S.-Q., Jordanova A., Kremensky I., Christodoulou K., Middleton L. T. et al. (2003). Glycyl tRNA synthetase mutations in Charcot-Marie-Tooth disease type 2D and distal spinal muscular atrophy type V. *Am. J. Hum. Genet.* 72, 1293-1299. 10.1086/375039 12690580 PMC1180282

[DMM028613C176] Aoki Y., Manzano R., Lee Y., Dafinca R., Aoki M., Douglas A. G. L., and Wood M. J. A. (2017). C9orf72 and RAB7L1 regulate vesicle trafficking in amyotrophic lateral sclerosis and frontotemporal dementia. *Brain* 140, 887-897. 10.1093/brain/awx024 28334866

[DMM028613C4] Ash P. E. A., Bieniek K. F., Gendron T. F., Caulfield T., Lin W.-L., DeJesus-Hernandez M., van Blitterswijk M. M., Jansen-West K., Paul J. W., Rademakers R. et al. (2013). Unconventional translation of C9ORF72 GGGGCC expansion generates insoluble polypeptides specific to c9FTD/ALS. *Neuron* 77, 639-646. 10.1016/j.neuron.2013.02.004 23415312 PMC3593233

[DMM028613C5] Barmada S. J., Skibinski G., Korb E., Rao E. J., Wu J. Y. and Finkbeiner S. (2010). Cytoplasmic mislocalization of TDP-43 is toxic to neurons and enhanced by a mutation associated with familial amyotrophic lateral sclerosis. *J. Neurosci.* 30, 639-649. 10.1523/JNEUROSCI.4988-09.2010 20071528 PMC2821110

[DMM028613C6] Barmada S. J., Serio A., Arjun A., Bilican B., Daub A., Ando D. M., Tsvetkov A., Pleiss M., Li X., Peisach D. et al. (2014). Autophagy induction enhances TDP43 turnover and survival in neuronal ALS models. *Nat. Chem. Biol.* 10, 677-685. 10.1038/nchembio.1563 24974230 PMC4106236

[DMM028613C7] Batra R., Charizanis K., Manchanda M., Mohan A., Li M., Finn D. J., Goodwin M., Zhang C., Sobczak K., Thornton C. A. et al. (2014). Loss of MBNL leads to disruption of developmentally regulated alternative polyadenylation in RNA-mediated disease. *Mol. Cell* 56, 311-322. 10.1016/j.molcel.2014.08.027 25263597 PMC4224598

[DMM028613C8] Belzil V. V., Gendron T. F. and Petrucelli L. (2013). RNA-mediated toxicity in neurodegenerative disease. *Mol. Cell. Neurosci.* 56, 406-419. 10.1016/j.mcn.2012.12.006 23280309 PMC3791208

[DMM028613C9] Bentmann E., Haass C. and Dormann D. (2013). Stress granules in neurodegeneration--lessons learnt from TAR DNA binding protein of 43 kDa and fused in sarcoma. *FEBS J.* 280, 4348-4370. 10.1111/febs.12287 23587065

[DMM028613C10] Bernard D., Prasanth K. V., Tripathi V., Colasse S., Nakamura T., Xuan Z., Zhang M. Q., Sedel F., Jourdren L., Coulpier F. et al. (2010). A long nuclear-retained non-coding RNA regulates synaptogenesis by modulating gene expression. *EMBO J.* 29, 3082-3093. 10.1038/emboj.2010.199 20729808 PMC2944070

[DMM028613C11] Broderick J. A. and Zamore P. D. (2011). MicroRNA therapeutics. *Gene Ther.* 18, 1104-1110. 10.1038/gt.2011.50 21525952 PMC3237828

[DMM028613C12] Brook J. D., McCurrach M. E., Harley H. G., Buckler A. J., Church D., Aburatani H., Hunter K., Stanton V. P., Thirion J.-P. and Hudson T. (1992). Molecular basis of myotonic dystrophy: expansion of a trinucleotide (CTG) repeat at the 3’ end of a transcript encoding a protein kinase family member. *Cell* 68, 799-808. 10.1016/0092-8674(92)90154-5 1310900

[DMM028613C13] Buratti E., Dörk T., Zuccato E., Pagani F., Romano M. and Baralle F. E. (2001). Nuclear factor TDP-43 and SR proteins promote in vitro and in vivo CFTR exon 9 skipping. *EMBO J.* 20, 1774-1784. 10.1093/emboj/20.7.1774 11285240 PMC145463

[DMM028613C14] Burberry A., Suzuki N., Wang J.-Y., Moccia R., Mordes D. A., Stewart M. H., Suzuki-Uematsu S., Ghosh S., Singh A., Merkle F. T. et al. (2016). Loss-of-function mutations in the C9ORF72 mouse ortholog cause fatal autoimmune disease. *Sci. Transl. Med.* 8, 347ra93 10.1126/scitranslmed.aaf6038 PMC5024536 27412785

[DMM028613C15] Butovsky O., Jedrychowski M. P., Cialic R., Krasemann S., Murugaiyan G., Fanek Z., Greco D. J., Wu P. M., Doykan C. E., Kiner O. et al. (2015). Targeting miR-155 restores abnormal microglia and attenuates disease in SOD1 mice. *Ann. Neurol.* 77, 75-99. 10.1002/ana.24304 25381879 PMC4432483

[DMM028613C16] Campuzano V., Montermini L., Molto M. D., Pianese L., Cossee M., Cavalcanti F., Monros E., Rodius F., Duclos F., Monticelli A. et al. (1996). Friedreich's ataxia: autosomal recessive disease caused by an intronic GAA triplet repeat expansion. *Science* 271, 1423-1427. 10.1126/science.271.5254.1423 8596916

[DMM028613C17] Cascella R., Capitini C., Fani G., Dobson C. M., Cecchi C. and Chiti F. (2016). Quantification of the relative contributions of loss-of-function and gain-of-function mechanisms in TAR DNA-binding protein 43 (TDP-43) proteinopathies. *J. Biol. Chem.* 291, 19437-19448. 10.1074/jbc.M116.737726 27445339 PMC5016682

[DMM028613C18] Cech T. R. and Steitz J. A. (2014). The noncoding RNA revolution-trashing old rules to forge new ones. *Cell* 157, 77-94. 10.1016/j.cell.2014.03.008 24679528

[DMM028613C19] Chen H.-J., Mitchell J., Novoselov S., Miller J., Nishimura A. L., Scotter E. L., Vance C. A., Cheetham M. E. and Shaw C. E. (2016). The heat shock response plays an important role in TDP-43 clearance: evidence for dysfunction in amyotrophic lateral sclerosis. *Brain* 139, 1417-1432. 10.1093/brain/aww028 26936937 PMC4845254

[DMM028613C20] Chen-Plotkin A. S., Unger T. L., Gallagher M. D., Bill E., Kwong L. K., Volpicelli-Daley L., Busch J. I., Akle S., Grossman M., Van Deerlin V. et al. (2012). TMEM106B, the risk gene for frontotemporal dementia, is regulated by the microRNA-132/212 cluster and affects progranulin pathways. *J. Neurosci.* 32, 11213-11227. 10.1523/JNEUROSCI.0521-12.2012 22895706 PMC3446826

[DMM028613C21] Chendrimada T. P., Gregory R. I., Kumaraswamy E., Norman J., Cooch N., Nishikura K. and Shiekhattar R. (2005). TRBP recruits the Dicer complex to Ago2 for microRNA processing and gene silencing. *Nature* 436, 740-744. 10.1038/nature03868 15973356 PMC2944926

[DMM028613C22] Ciura S., Lattante S., Le Ber I., Latouche M., Tostivint H., Brice A. and Kabashi E. (2013). Loss of function of C9orf72 causes motor deficits in a zebrafish model of amyotrophic lateral sclerosis. *Ann. Neurol.* 74, 180-187. 10.1002/ana.23946 23720273

[DMM028613C23] Cogswell J. P., Ward J., Taylor I. A., Waters M., Shi Y., Cannon B., Kelnar K., Kemppainen J., Brown D., Chen C. et al. (2008). Identification of miRNA changes in Alzheimer's disease brain and CSF yields putative biomarkers and insights into disease pathways. *J. Alzheimers Dis.* 14, 27-41. 10.3233/JAD-2008-14103 18525125

[DMM028613C24] Cooper-Knock J., Walsh M. J., Higginbottom A., Robin Highley J., Dickman M. J., Edbauer D., Ince P. G., Wharton S. B., Wilson S. A., Kirby J. et al. (2014). Sequestration of multiple RNA recognition motif-containing proteins by C9orf72 repeat expansions. *Brain* 137, 2040-2051. 10.1093/brain/awu120 24866055 PMC4065024

[DMM028613C25] Costessi L., Porro F., Iaconcig A. and Muro A. F. (2014). TDP-43 regulates β-adducin (Add2) transcript stability. *RNA Biol.* 11, 1280-1290. 10.1080/15476286.2014.996081 25602706 PMC4615836

[DMM028613C26] Courchaine E. M., Lu A. and Neugebauer K. M. (2016). Droplet organelles? *EMBO J.* 35, 1603-1612. 10.15252/embj.201593517 27357569 PMC4969579

[DMM028613C27] Cui J. G., Li Y. Y., Zhao Y., Bhattacharjee S. and Lukiw W. J. (2010). Differential regulation of interleukin-1 receptor-associated kinase-1 (IRAK-1) and IRAK-2 by microRNA-146a and NF-kappaB in stressed human astroglial cells and in Alzheimer disease. *J. Biol. Chem.* 285, 38951-38960. 10.1074/jbc.M110.178848 20937840 PMC2998119

[DMM028613C28] Daughters R. S., Tuttle D. L., Gao W., Ikeda Y., Moseley M. L., Ebner T. J., Swanson M. S. and Ranum L. P. (2009). RNA gain-of-function in spinocerebellar ataxia type 8. *PLoS Genet.* 5, e1000600 10.1371/journal.pgen.1000600 19680539 PMC2719092

[DMM028613C29] Davis B. M., McCurrach M. E., Taneja K. L., Singer R. H. and Housman D. E. (1997). Expansion of a CUG trinucleotide repeat in the 3′ untranslated region of myotonic dystrophy protein kinase transcripts results in nuclear retention of transcripts. *Proc. Natl. Acad. Sci. USA* 94, 7388-7393. 10.1073/pnas.94.14.7388 9207101 PMC23831

[DMM028613C30] De Felice B., Annunziata A., Fiorentino G., Borra M., Biffali E., Coppola C., Cotrufo R., Brettschneider J., Giordana M. L., Dalmay T. et al. (2014). miR-338-3p is over-expressed in blood, CFS, serum and spinal cord from sporadic amyotrophic lateral sclerosis patients. *Neurogenetics* 15, 243-253. 10.1007/s10048-014-0420-2 25130371

[DMM028613C31] DeJesus-Hernandez M., Mackenzie I. R., Boeve B. F., Boxer A. L., Baker M., Rutherford N. J., Nicholson A. M., Finch N. A., Flynn H., Adamson J. et al. (2011). Expanded GGGGCC hexanucleotide repeat in noncoding region of C9ORF72 causes chromosome 9p-linked FTD and ALS. *Neuron* 72, 245-256. 10.1016/j.neuron.2011.09.011 21944778 PMC3202986

[DMM028613C32] Disney M. D., Liu B., Yang W.-Y., Sellier C., Tran T., Charlet-Berguerand N. and Childs-Disney J. L. (2012). A small molecule that targets r(CGG)(exp) and improves defects in fragile X-associated tremor ataxia syndrome. *ACS Chem. Biol.* 7, 1711-1718. 10.1021/cb300135h 22948243 PMC3477254

[DMM028613C33] Donnelly C. J., Zhang P., Pham J. T., Heusler A. R., Mistry N. A., Vidensky S., Daley E. L., Poth E. M., Fines D. M., Maragakis N. et al. (2013). RNA toxicity from the ALS/FTD C9ORF72 expansion is mitigated by antisense intervention. *Neuron* 80, 415-428. 10.1016/j.neuron.2013.10.015 24139042 PMC4098943

[DMM028613C34] Durr A. (2010). Autosomal dominant cerebellar ataxias: polyglutamine expansions and beyond. *Lancet Neurol.* 9, 885-894. 10.1016/S1474-4422(10)70183-6 20723845

[DMM028613C35] Elden A. C., Kim H.-J. J., Hart M. P., Chen-Plotkin A. S., Johnson B. S., Fang X., Armakola M., Geser F., Greene R., Lu M. M. et al. (2010). Ataxin-2 intermediate-length polyglutamine expansions are associated with increased risk for ALS. *Nature* 466, 1069-1075. 10.1038/nature09320 20740007 PMC2965417

[DMM028613C36] Emde A., Eitan C., Liou L.-L., Libby R. T., Rivkin N., Magen I., Reichenstein I., Oppenheim H., Eilam R., Silvestroni A. et al. (2015). Dysregulated miRNA biogenesis downstream of cellular stress and ALS-causing mutations: a new mechanism for ALS. *EMBO J.* 34, 2633-2651. 10.15252/embj.201490493 26330466 PMC4641530

[DMM028613C37] Faghihi M. A., Modarresi F., Khalil A. M., Wood D. E., Sahagan B. G., Morgan T. E., Finch C. E., St. Laurent G., Kenny P. and Wahlestedt C. (2008). Expression of a noncoding RNA is elevated in Alzheimer's disease and drives rapid feed-forward regulation of beta-secretase. *Nat. Med.* 14, 723-730. 10.1038/nm1784 18587408 PMC2826895

[DMM028613C38] Fatemi R. P., Velmeshev D. and Faghihi M. A. (2014). De-repressing LncRNA-targeted genes to upregulate gene expression: focus on small molecule therapeutics. *Mol. Ther. Nucleic Acids* 3, e196 10.1038/mtna.2014.45 25405465 PMC4461991

[DMM028613C39] Figueroa-Romero C., Hur J., Lunn J. S., Paez-Colasante X., Bender D. E., Yung R., Sakowski S. A. and Feldman E. L. (2016). Expression of microRNAs in human post-mortem amyotrophic lateral sclerosis spinal cords provides insight into disease mechanisms. *Mol. Cell. Neurosci.* 71, 34-45. 10.1016/j.mcn.2015.12.008 26704906 PMC4761498

[DMM028613C40] Fox A. H. and Lamond A. I. (2010). Paraspeckles. *Cold Spring Harbor Perspect. Biol.* 2, a000687 10.1101/cshperspect.a000687 PMC2890200 20573717

[DMM028613C41] Fu H., Feng J., Liu Q., Sun F., Tie F., Zhu J., Xing R., Sun Z. and Zheng X. (2009). Stress induces tRNA cleavage by angiogenin in mammalian cells. *FEBS Lett.* 583, 437-442. 10.1016/j.febslet.2008.12.043 19114040

[DMM028613C42] Gaughwin P. M., Ciesla M., Lahiri N., Tabrizi S. J., Brundin P. and Björkqvist M. (2011). Hsa-miR-34b is a plasma-stable microRNA that is elevated in pre-manifest Huntington's disease. *Hum. Mol. Genet.* 20, 2225-2237. 10.1093/hmg/ddr111 21421997

[DMM028613C43] GBD 2015 Mortality and Causes of Death Collaborators. (2016). Global, regional, and national life expectancy, all-cause mortality, and cause-specific mortality for 249 causes of death, 1980-2015: a systematic analysis for the Global Burden of Disease Study 2015. *Lancet* 388, 1459-1544. 10.1016/S0140-6736(16)31012-1 27733281 PMC5388903

[DMM028613C44] Gendron T. F., Bieniek K. F., Zhang Y.-J., Jansen-West K., Ash P. E. A., Caulfield T., Daughrity L., Dunmore J. H., Castanedes-Casey M., Chew J. et al. (2013). Antisense transcripts of the expanded C9ORF72 hexanucleotide repeat form nuclear RNA foci and undergo repeat-associated non-ATG translation in c9FTD/ALS. *Acta Neuropathol.* 126, 829-844. 10.1007/s00401-013-1192-8 24129584 PMC3830741

[DMM028613C45] Gitcho M. A., Baloh R. H., Chakraverty S., Mayo K., Norton J. B., Levitch D., Hatanpaa K. J., White C. L., Bigio E. H., Caselli R. et al. (2008). TDP-43 A315T mutation in familial motor neuron disease. *Ann. Neurol.* 63, 535-538. 10.1002/ana.21344 18288693 PMC2747362

[DMM028613C46] Goodwin M., Mohan A., Batra R., Lee K.-Y., Charizanis K., Gómez F. J. F., Eddarkaoui S., Sergeant N., Buée L., Kimura T. et al. (2015). MBNL sequestration by toxic RNAs and RNA misprocessing in the myotonic dystrophy brain. *Cell Rep.* 12, 1159-1168. 10.1016/j.celrep.2015.07.029 26257173 PMC4545389

[DMM028613C47] Grabczyk E. and Usdin K. (2000). The GAATTC triplet repeat expanded in Friedreich's ataxia impedes transcription elongation by T7 RNA polymerase in a length and supercoil dependent manner. *Nucleic Acids Res.* 28, 2815-2822. 10.1093/nar/28.14.2815 10908340 PMC102661

[DMM028613C48] Greenway M. J., Alexander M. D., Ennis S., Traynor B. J., Corr B., Frost E., Green A. and Hardiman O. (2004). A novel candidate region for ALS on chromosome 14q11. 2. *Neurology* 63, 1936-1938. 10.1212/01.WNL.0000144344.39103.F6 15557516

[DMM028613C49] Greenway M. J., Andersen P. M., Russ C., Ennis S., Cashman S., Donaghy C., Patterson V., Swingler R., Kieran D., Prehn J. et al. (2006). ANG mutations segregate with familial and'sporadic'amyotrophic lateral sclerosis. *Nat. Genet.* 38, 411-413. 10.1038/ng1742 16501576

[DMM028613C50] Guo L. and Shorter J. (2015). It's raining liquids: RNA tunes viscoelasticity and dynamics of membraneless organelles. *Mol. Cell* 60, 189-192. 10.1016/j.molcel.2015.10.006 26474062 PMC4653132

[DMM028613C51] Halliday G., Bigio E. H., Cairns N. J., Neumann M., Mackenzie I. R. A. and Mann D. M. A. (2012). Mechanisms of disease in frontotemporal lobar degeneration: gain of function versus loss of function effects. *Acta Neuropathol.* 124, 373-382. 10.1007/s00401-012-1030-4 22878865 PMC3445027

[DMM028613C52] Hardy J. and Selkoe D. (2002). The amyloid hypothesis of Alzheimer's disease: progress and problems on the road to therapeutics. *Science* 297, 353-356. 10.1126/science.1072994 12130773

[DMM028613C53] Hébert S. S., Horré K., Nicolaï L., Papadopoulou A. S., Mandemakers W., Silahtaroglu A. N., Kauppinen S., Delacourte A. and Strooper B. (2008). Loss of microRNA cluster miR-29a/b-1 in sporadic Alzheimer's disease correlates with increased BACE1/beta-secretase expression. *Proc. Natl. Acad. Sci. USA* 105, 6415-6420. 10.1073/pnas.0710263105 18434550 PMC2359789

[DMM028613C54] Hoss A. G., Labadorf A., Latourelle J. C., Kartha V. K., Hadzi T. C., Gusella J. F., MacDonald M. E., Chen J.-F. F., Akbarian S., Weng Z. et al. (2015). miR-10b-5p expression in Huntington's disease brain relates to age of onset and the extent of striatal involvement. *BMC Med. Genomics* 8, 10 10.1186/s12920-015-0083-3 25889241 PMC4349621

[DMM028613C55] Huang C., Zhou H., Tong J., Chen H., Liu Y.-J., Wang D., Wei X. and Xia X.-G. (2011). FUS transgenic rats develop the phenotypes of amyotrophic lateral sclerosis and frontotemporal lobar degeneration. *PLoS Genet.* 7, e1002011 10.1371/journal.pgen.1002011 21408206 PMC3048370

[DMM028613C56] Huber K. M., Gallagher S. M., Warren S. T. and Bear M. F. (2002). Altered synaptic plasticity in a mouse model of fragile X mental retardation. *Proc. Natl. Acad. Sci. USA* 99, 7746-7750. 10.1073/pnas.122205699 12032354 PMC124340

[DMM028613C57] Igaz L. M., Kwong L. K., Lee E. B., Chen-Plotkin A., Swanson E., Unger T., Malunda J., Xu Y., Winton M. J., Trojanowski J. Q. et al. (2011). Dysregulation of the ALS-associated gene TDP-43 leads to neuronal death and degeneration in mice. *J. Clin. Invest.* 121, 726-738. 10.1172/JCI44867 21206091 PMC3026736

[DMM028613C58] Ishimura R., Nagy G., Dotu I., Zhou H., Yang X.-L., Schimmel P., Senju S., Nishimura Y., Chuang J. and Ackerman S. (2014). Ribosome stalling induced by mutation of a CNS-specific tRNA causes neurodegeneration. *Science* 345, 455-459. 10.1126/science.1249749 25061210 PMC4281038

[DMM028613C59] Ivanov P., Emara M. M., Villen, J., Gygi S. P. and Anderson P. (2011). Angiogenin-induced tRNA fragments inhibit translation initiation. *Mol. Cell* 43, 613-623. 10.1016/j.molcel.2011.06.022 21855800 PMC3160621

[DMM028613C60] Iwahashi C. K., Yasui D. H., An H.-J., Greco C. M., Tassone F., Nannen K., Babineau B., Lebrilla C. B., Hagerman R. J. and Hagerman P. J. (2006). Protein composition of the intranuclear inclusions of FXTAS. *Brain* 129, 256-271. 10.1093/brain/awh650 16246864

[DMM028613C61] Iyer A., Zurolo E., Prabowo A., Fluiter K., Spliet W. G., van Rijen P. C., Gorter J. A. and Aronica E. (2012). MicroRNA-146a: a key regulator of astrocyte-mediated inflammatory response. *PLoS ONE* 7, e44789 10.1371/journal.pone.0044789 23028621 PMC3441440

[DMM028613C62] Jackrel M., DeSantis M., Martinez B., Castellano L., Stewart R., Caldwell K., Caldwell G. and Shorter J. (2014). Potentiated Hsp104 variants antagonize diverse proteotoxic misfolding events. *Cell* 156, 170-182. 10.1016/j.cell.2013.11.047 24439375 PMC3909490

[DMM028613C63] Jiang J. and Cleveland D. W. (2016). Bidirectional transcriptional inhibition as therapy for ALS/FTD caused by repeat expansion in C9orf72. *Neuron* 92, 1160-1163. 10.1016/j.neuron.2016.12.008 28009271

[DMM028613C64] Jiang H., Mankodi A., Swanson M. S., Moxley R. T. and Thornton C. A. (2004). Myotonic dystrophy type 1 is associated with nuclear foci of mutant RNA, sequestration of muscleblind proteins and deregulated alternative splicing in neurons. *Hum. Mol. Genet.* 13, 3079-3088. 10.1093/hmg/ddh327 15496431

[DMM028613C65] Jiang J., Zhu Q., Gendron T., Saberi S., McAlonis-Downes M., Seelman A., Stauffer J., Jafar-Nejad P., Drenner K., Schulte D. et al. (2016). Gain of toxicity from ALS/FTD-linked repeat expansions in C9ORF72 is alleviated by antisense oligonucleotides targeting GGGGCC-containing RNAs. *Neuron* 90, 535-550. 10.1016/j.neuron.2016.04.006 27112497 PMC4860075

[DMM028613C66] Jin P., Duan R., Qurashi A., Qin Y., Tian D., Rosser T. C., Liu H., Feng Y. and Warren S. T. (2007). Pur alpha binds to rCGG repeats and modulates repeat-mediated neurodegeneration in a Drosophila model of fragile X tremor/ataxia syndrome. *Neuron* 55, 556-564. 10.1016/j.neuron.2007.07.020 17698009 PMC1994817

[DMM028613C67] Johnson R. (2012). Long non-coding RNAs in Huntington's disease neurodegeneration. *Neurobiol. Dis.* 46, 245-254. 10.1016/j.nbd.2011.12.006 22202438

[DMM028613C68] Johnson R. and Buckley N. J. (2009). Gene dysregulation in Huntington's disease: REST, microRNAs and beyond. *Neuromolecular Med.* 11, 183-199. 10.1007/s12017-009-8063-4 19458943

[DMM028613C69] Johnson B. S., McCaffery J. M., Lindquist S. and Gitler A. D. (2008). A yeast TDP-43 proteinopathy model: exploring the molecular determinants of TDP-43 aggregation and cellular toxicity. 105, 6439-6444. 10.1073/pnas.0802082105 PMC2359814 18434538

[DMM028613C70] Johnson B. S., Snead D., Lee J. J., McCaffery J. M., Shorter J. and Gitler A. D. (2009). TDP-43 is intrinsically aggregation-prone, and amyotrophic lateral sclerosis-linked mutations accelerate aggregation and increase toxicity. *J. Biol. Chem.* 284, 20329-20339. 10.1074/jbc.M109.010264 19465477 PMC2740458

[DMM028613C71] Jovicic A., Zaldivar Jolissaint J. F., Moser R., Silva Santos M. de F. and Luthi-Carter R. (2013). MicroRNA-22 (miR-22) overexpression is neuroprotective via general anti-apoptotic effects and may also target specific Huntington's disease-related mechanisms. *PLoS ONE* 8, e54222 10.1371/journal.pone.0054222 23349832 PMC3547907

[DMM028613C72] Kabashi E., Valdmanis P. N., Dion P., Spiegelman D., McConkey B. J., Vande V. C., Bouchard J.-P. P., Lacomblez L., Pochigaeva K., Salachas F. et al. (2008). TARDBP mutations in individuals with sporadic and familial amyotrophic lateral sclerosis. *Nat. Genet.* 40, 572-574. 10.1038/ng.132 18372902

[DMM028613C73] Karaca E., Weitzer S., Pehlivan D., Shiraishi H., Gogakos T., Hanada T., Jhangiani S. N., Wiszniewski W., Withers M., Campbell I. M. et al. (2014). Human CLP1 mutations alter tRNA biogenesis, affecting both peripheral and central nervous system function. *Cell* 157, 636-650. 10.1016/j.cell.2014.02.058 24766809 PMC4146440

[DMM028613C74] Kato M., Han T. W., Xie S., Shi K., Du X., Wu L. C., Mirzaei H., Goldsmith E. J., Longgood J., Pei J. et al. (2012). Cell-free formation of RNA granules: low complexity sequence domains form dynamic fibers within hydrogels. *Cell* 149, 753-767. 10.1016/j.cell.2012.04.017 22579281 PMC6347373

[DMM028613C75] Kawahara Y. and Mieda-Sato A. (2012). TDP-43 promotes microRNA biogenesis as a component of the Drosha and Dicer complexes. *Proc. Natl. Acad. Sci. USA* 109, 3347-3352. 10.1073/pnas.1112427109 22323604 PMC3295278

[DMM028613C76] Khalil A. M., Guttman M., Huarte M., Garber M., Raj A., Morales D., Thomas K., Presser A., Bernstein B. E., Oudenaarden A. et al. (2009). Many human large intergenic noncoding RNAs associate with chromatin-modifying complexes and affect gene expression. *Proc. Natl. Acad. Sci. USA* 106, 11667-11672. 10.1073/pnas.0904715106 19571010 PMC2704857

[DMM028613C77] Kim J., Inoue K., Ishii J., Vanti W. B., Voronov S. V., Murchison E., Hannon G. and Abeliovich A. (2007). A MicroRNA feedback circuit in midbrain dopamine neurons. *Science* 317, 1220-1224. 10.1126/science.1140481 17761882 PMC2782470

[DMM028613C78] Kim H. J., Kim N. C., Wang Y.-D., Scarborough E. A., Moore J., Diaz Z., MacLea K. S., Freibaum B., Li S., Molliex A. et al. (2013). Mutations in prion-like domains in hnRNPA2B1 and hnRNPA1 cause multisystem proteinopathy and ALS. *Nature* 495, 467-473. 10.1038/nature11922 23455423 PMC3756911

[DMM028613C79] Kim W., Lee Y., McKenna N. D., Yi M., Simunovic F., Wang Y., Kong B., Rooney R. J., Seo H., Stephens R. M. et al. (2014). miR-126 contributes to Parkinson's disease by dysregulating the insulin-like growth factor/phosphoinositide 3-kinase signaling. *Neurobiol. Aging* 35, 1712-1721. 10.1016/j.neurobiolaging.2014.01.021 24559646 PMC3991567

[DMM028613C80] Kiriakidou M., Tan G. S., Lamprinaki S., De Planell-Saguer M., Nelson P. T. and Mourelatos Z. (2007). An mRNA m7G cap binding-like motif within human Ago2 represses translation. *Cell* 129, 1141-1151. 10.1016/j.cell.2007.05.016 17524464

[DMM028613C81] Kordasiewicz H. B., Stanek L. M., Wancewicz E. V., Mazur C., McAlonis M. M., Pytel K. A., Artates J. W., Weiss A., Cheng S. H., Shihabuddin L. S. et al. (2012). Sustained therapeutic reversal of Huntington's disease by transient repression of huntingtin synthesis. *Neuron* 74, 1031-1044. 10.1016/j.neuron.2012.05.009 22726834 PMC3383626

[DMM028613C82] Kramer N. J., Carlomagno Y., Zhang Y.-J. J., Almeida S., Cook C. N., Gendron T. F., Prudencio M., Van Blitterswijk M., Belzil V., Couthouis J. et al. (2016). Spt4 selectively regulates the expression of C9orf72 sense and antisense mutant transcripts. *Science* 353, 708-712. 10.1126/science.aaf7791 27516603 PMC5823025

[DMM028613C83] Kroschwald S., Maharana S., Mateju D., Malinovska L., Nüske E., Poser I., Richter D. and Alberti S. (2015). Promiscuous interactions and protein disaggregases determine the material state of stress-inducible RNP granules. *Elife* 4, e06807 10.7554/eLife.06807 26238190 PMC4522596

[DMM028613C84] Kwiatkowski T. J., Bosco D. A., LeClerc A. L., Tamrazian E., Vanderburg C. R., Russ C., Davis A., Gilchrist J., Kasarskis E. J., Munsat T. et al. (2009). Mutations in the FUS/TLS gene on chromosome 16 cause familial amyotrophic lateral sclerosis. *Science* 323, 1205-1208. 10.1126/science.1166066 19251627

[DMM028613C85] Lagier-Tourenne C., Polymenidou M., Hutt K. R., Vu A. Q., Baughn M., Huelga S. C., Clutario K. M., Ling S.-C., Liang T. Y., Mazur C. et al. (2012). Divergent roles of ALS-linked proteins FUS/TLS and TDP-43 intersect in processing long pre-mRNAs. *Nat. Neurosci.* 15, 1488-1497. 10.1038/nn.3230 23023293 PMC3586380

[DMM028613C86] Lagier-Tourenne C., Baughn M., Rigo F., Sun S., Liu P., Li H.-R., Jiang J., Watt A. T., Chun S., Katz M. et al. (2013). Targeted degradation of sense and antisense C9orf72 RNA foci as therapy for ALS and frontotemporal degeneration. *Proc. Natl. Acad. Sci. USA* 110, E4530-E4539. 10.1073/pnas.1318835110 24170860 PMC3839752

[DMM028613C87] Langa K. M., Larson E. B., Crimmins E. M., Faul J. D., Levine D. A., Kabeto M. U. and Weir D. R. (2017). A Comparison of the prevalence of dementia in the united states in 2000 and 2012. *JAMA Intern. Med.* 177, 51-58. 10.1001/jamainternmed.2016.6807 27893041 PMC5195883

[DMM028613C88] Lau P., Bossers K., Janky R., Salta E., Frigerio C. S., Barbash S., Rothman R., Sierksma A. S. R., Thathiah A., Greenberg D. et al. (2013). Alteration of the microRNA network during the progression of Alzheimer's disease. *EMBO Mol. Med.* 5, 1613-1634. 10.1002/emmm.201201974 24014289 PMC3799583

[DMM028613C89] Lee Y., Ahn C., Han J., Choi H., Kim J., Yim J., Lee J., Provost P., Rådmark O., Kim S. et al. (2003). The nuclear RNase III Drosha initiates microRNA processing. *Nature* 425, 415-419. 10.1038/nature01957 14508493

[DMM028613C90] Lee S.-T. T., Chu K., Jung K.-H. H., Kim J. H., Huh J.-Y. Y., Yoon H., Park D.-K. K., Lim J.-Y. Y., Kim J.-M. M., Jeon D. et al. (2012). miR-206 regulates brain-derived neurotrophic factor in Alzheimer disease model. *Ann. Neurol.* 72, 269-277. 10.1002/ana.23588 22926857

[DMM028613C91] Lee Y.-B., Chen H.-J., Peres J. N., Gomez-Deza J., Attig J., Štalekar M., Troakes C., Nishimura A. L., Scotter E. L., Vance C. et al. (2013). Hexanucleotide repeats in ALS/FTD form length-dependent RNA foci, sequester RNA binding proteins, and are neurotoxic. *Cell Rep.* 5, 1178-1186. 10.1016/j.celrep.2013.10.049 24290757 PMC3898469

[DMM028613C92] Lehmann S. M., Krüger C., Park B., Derkow K., Rosenberger K., Baumgart J., Trimbuch T., Eom G., Hinz M., Kaul D. et al. (2012). An unconventional role for miRNA: let-7 activates Toll-like receptor 7 and causes neurodegeneration. *Nat. Neurosci.* 15, 827-835. 10.1038/nn.3113 22610069

[DMM028613C93] Li Y. R., King O. D., Shorter J. and Gitler A. D. (2013). Stress granules as crucibles of ALS pathogenesis. *J. Cell Biol.* 201, 361-372. 10.1083/jcb.201302044 23629963 PMC3639398

[DMM028613C94] Lin Y., Protter D. S., Rosen M. K. and Parker R. (2015). Formation and maturation of phase-separated liquid droplets by RNA-binding proteins. *Mol. Cell* 60, 208-219. 10.1016/j.molcel.2015.08.018 26412307 PMC4609299

[DMM028613C95] Lindquist S. and Craig E. A. (1988). The heat-shock proteins. *Annu. Rev. Genet.* 22, 631-677. 10.1146/annurev.ge.22.120188.003215 2853609

[DMM028613C96] Ling S.-C., Albuquerque C. P., Han J. S., Lagier-Tourenne C., Tokunaga S., Zhou H. and Cleveland D. W. (2010). ALS-associated mutations in TDP-43 increase its stability and promote TDP-43 complexes with FUS/TLS. *Proc. Natl. Acad. Sci. USA* 107, 13318-13323. 10.1073/pnas.1008227107 20624952 PMC2922163

[DMM028613C97] Ling S.-C., Polymenidou M. and Cleveland D. W. (2013). Converging mechanisms in ALS and FTD: disrupted RNA and protein homeostasis. *Neuron* 79, 416-438. 10.1016/j.neuron.2013.07.033 23931993 PMC4411085

[DMM028613C98] Ling J. P., Pletnikova O., Troncoso J. C. and Wong P. C. (2015). TDP-43 repression of nonconserved cryptic exons is compromised in ALS-FTD. *Science* 349, 650-655. 10.1126/science.aab0983 26250685 PMC4825810

[DMM028613C99] Liquori C. L., Ricker K., Moseley M. L., Jacobsen J. F., Kress W., Naylor S. L., Day J. W. and Ranum L. P. (2001). Myotonic dystrophy type 2 caused by a CCTG expansion in intron 1 of ZNF9. *Science* 293, 864-867. 10.1126/science.1062125 11486088

[DMM028613C100] Liu X., Li D., Zhang W., Guo M. and Zhan Q. (2012). Long non-coding RNA gadd7 interacts with TDP-43 and regulates Cdk6 mRNA decay. *EMBO J.* 31, 4415-4427. 10.1038/emboj.2012.292 23103768 PMC3512391

[DMM028613C101] Liu E., Russ J., Wu K., Neal D., Suh E., McNally A., Irwin D. J., Van Deerlin V. M. and Lee E. B. (2014). C9orf72 hypermethylation protects against repeat expansion-associated pathology in ALS/FTD. *Acta Neuropathol.* 128, 525-541. 10.1007/s00401-014-1286-y 24806409 PMC4161616

[DMM028613C102] Lokireddy S., Kukushkin N. and Goldberg A. (2015). cAMP-induced phosphorylation of 26S proteasomes on Rpn6/PSMD11 enhances their activity and the degradation of misfolded proteins. *Proc. Natl Acad. Sci. USA* 112, E7176-E7185. 10.1073/pnas.1522332112 26669444 PMC4702992

[DMM028613C103] Loureiro J. R., Oliveira C. L. and Silveira I. (2016). Unstable repeat expansions in neurodegenerative diseases: nucleocytoplasmic transport emerges on the scene. *Neurobiol. Aging* 39, 174-183. 10.1016/j.neurobiolaging.2015.12.007 26923414

[DMM028613C104] Lukiw W. J. and Alexandrov P. N. (2012). Regulation of complement factor H (CFH) by multiple miRNAs in Alzheimer's disease (AD) brain. *Mol. Neurobiol.* 46, 11-19. 10.1007/s12035-012-8234-4 22302353 PMC3703615

[DMM028613C105] Luu L. M., Nguyen L., Peng S., Lee J., Lee H. Y., Wong C.-H., Hergenrother P. J., Chan H. Y. E. and Zimmerman S. C. (2016). A potent inhibitor of protein sequestration by expanded triplet (CUG) repeats that shows phenotypic improvements in a drosophila model of myotonic dystrophy. *ChemMedChem* 11, 1428-1435. 10.1002/cmdc.201600081 27245480 PMC5074844

[DMM028613C106] MacDonald M. E., Barnes G., Srinidhi J., Duyao M. P., Ambrose C. M., Myers R. H., Gray J., Conneally P. M., Young A. and Penney J. (1993). Gametic but not somatic instability of CAG repeat length in Huntington's disease. *J. Med. Genet.* 30, 982-986. 10.1136/jmg.30.12.982 8133508 PMC1016628

[DMM028613C107] Mangiarini L., Sathasivam K., Seller M., Cozens B., Harper A., Hetherington C., Lawton M., Trottier Y., Lehrach H., Davies S. W. et al. (1996). Exon 1 of the HD gene with an expanded CAG repeat is sufficient to cause a progressive neurological phenotype in transgenic mice. *Cell* 87, 493-506. 10.1016/S0092-8674(00)81369-0 8898202

[DMM028613C108] McMillan C. T., Russ J., Wood E. M., Irwin D. J., Grossman M., McCluskey L., Elman L., Van Deerlin V. and Lee E. B. (2015). C9orf72 promoter hypermethylation is neuroprotective: neuroimaging and neuropathologic evidence. *Neurology* 84, 1622-1630. 10.1212/WNL.0000000000001495 25795648 PMC4409587

[DMM028613C109] Miller J. W., Urbinati C. R., Teng-Umnuay P., Stenberg M. G., Byrne B. J., Thornton C. A. and Swanson M. S. (2000). Recruitment of human muscleblind proteins to (CUG)*_n_* expansions associated with myotonic dystrophy. *EMBO J.* 19, 4439-4448. 10.1093/emboj/19.17.4439 10970838 PMC302046

[DMM028613C110] Miñones-Moyano E., Porta S., Escaramís G., Rabionet R., Iraola S., Kagerbauer B., Espinosa-Parrilla Y., Ferrer I., Estivill X. and Martí E. (2011). MicroRNA profiling of Parkinson's disease brains identifies early downregulation of miR-34b/c which modulate mitochondrial function. *Hum. Mol. Genet.* 20, 3067-3078. 10.1093/hmg/ddr210 21558425

[DMM028613C111] Molliex A., Temirov J., Lee J., Coughlin M., Kanagaraj A. P., Kim H. J., Mittag T. and Taylor J. P. (2015). Phase separation by low complexity domains promotes stress granule assembly and drives pathological fibrillization. *Cell* 163, 123-133. 10.1016/j.cell.2015.09.015 26406374 PMC5149108

[DMM028613C112] Moseley M. L., Zu T., Ikeda Y., Gao W., Mosemiller A. K., Daughters R. S., Chen G., Weatherspoon M. R., Clark H. B., Ebner T. J. et al. (2006). Bidirectional expression of CUG and CAG expansion transcripts and intranuclear polyglutamine inclusions in spinocerebellar ataxia type 8. *Nat. Genet.* 38, 758-769. 10.1038/ng1827 16804541

[DMM028613C113] Motley W. W., Talbot K. and Fischbeck K. H. (2010). GARS axonopathy: not every neuron's cup of tRNA. *Trends Neurosci.* 33, 59-66. 10.1016/j.tins.2009.11.001 20152552 PMC2822721

[DMM028613C114] Murakami T., Qamar S., Lin J. Q., Schierle G. S., Rees E., Miyashita A., Costa A. R., Dodd R. B., Chan F. T. S., Michel C. H. et al. (2015). ALS/FTD mutation-induced phase transition of FUS liquid droplets and reversible hydrogels into irreversible hydrogels impairs RNP granule function. *Neuron* 88, 678-690. 10.1016/j.neuron.2015.10.030 26526393 PMC4660210

[DMM028613C115] Nangle L. A., Zhang W., Xie W., Yang X.-L. and Schimmel P. (2007). Charcot-Marie-Tooth disease-associated mutant tRNA synthetases linked to altered dimer interface and neurite distribution defect. *Proc. Natl. Acad. Sci. USA* 104, 11239-11244. 10.1073/pnas.0705055104 17595294 PMC2040883

[DMM028613C116] Nelson P. T. and Wang W.-X. X. (2010). MiR-107 is reduced in Alzheimer's disease brain neocortex: validation study. *J. Alzheimers Dis.* 21, 75-79. 10.3233/JAD-2010-091603 20413881 PMC2910235

[DMM028613C117] Neumann M., Sampathu D. M., Kwong L. K., Truax A. C., Micsenyi M. C., Chou T. T., Bruce J., Schuck T., Grossman M., Clark C. M. et al. (2006). Ubiquitinated TDP-43 in frontotemporal lobar degeneration and amyotrophic lateral sclerosis. *Science* 314, 130-133. 10.1126/science.1134108 17023659

[DMM028613C118] Niehues S., Bussmann J., Steffes G., Erdmann I., Köhrer C., Sun L., Wagner M., Schäfer K., Wang G., Koerdt S. N. et al. (2015). Impaired protein translation in Drosophila models for Charcot-Marie-Tooth neuropathy caused by mutant tRNA synthetases. *Nat. Commun.* 6, 7520 10.1038/ncomms8520 26138142 PMC4506996

[DMM028613C119] Oh S. Y., He F., Krans A., Frazer M., Taylor J. P., Paulson H. L. and Todd P. K. (2015). RAN translation at CGG repeats induces ubiquitin proteasome system impairment in models of fragile X-associated tremor ataxia syndrome. *Hum. Mol. Genet.* 24, 4317-4326. 10.1093/hmg/ddv165 25954027 PMC4492395

[DMM028613C120] Packer A. N., Xing Y., Harper S. Q., Jones L. and Davidson B. L. (2008). The bifunctional microRNA miR-9/miR-9* regulates REST and CoREST and is downregulated in Huntington's disease. *J. Neurosci.* 28, 14341-14346. 10.1523/JNEUROSCI.2390-08.2008 19118166 PMC3124002

[DMM028613C121] Paushkin S. V., Patel M., Furia B. S., Peltz S. W. and Trotta C. R. (2004). Identification of a human endonuclease complex reveals a link between tRNA splicing and pre-mRNA 3′ end formation. *Cell* 117, 311-321. 10.1016/S0092-8674(04)00342-3 15109492

[DMM028613C122] Pieretti M., Zhang F. P., Fu Y.-H., Warren S. T., Oostra B. A., Caskey C. T. and Nelson D. L. (1991). Absence of expression of the FMR-1 gene in fragile X syndrome. *Cell* 66, 817-822. 10.1016/0092-8674(91)90125-I 1878973

[DMM028613C123] Polymenidou M., Lagier-Tourenne C., Hutt K. R., Huelga S. C., Moran J., Liang T. Y., Ling S.-C. C., Sun E., Wancewicz E., Mazur C. et al. (2011). Long pre-mRNA depletion and RNA missplicing contribute to neuronal vulnerability from loss of TDP-43. *Nat. Neurosci.* 14, 459-468. 10.1038/nn.2779 21358643 PMC3094729

[DMM028613C124] Prensner J. R. and Chinnaiyan A. M. (2011). The emergence of lncRNAs in cancer biology. *Cancer Discov.* 1, 391-407. 10.1158/2159-8290.CD-11-0209 22096659 PMC3215093

[DMM028613C125] Renton A. E., Majounie E., Waite A., Simón-Sánchez J., Rollinson S., Gibbs J. R., Schymick J. C., Laaksovirta H., van Swieten J. C., Myllykangas L. et al. (2011). A hexanucleotide repeat expansion in C9ORF72 is the cause of chromosome 9p21-linked ALS-FTD. *Neuron* 72, 257-268. 10.1016/j.neuron.2011.09.010 21944779 PMC3200438

[DMM028613C126] Russ J., Liu E. Y., Wu K., Neal D., Suh E., Irwin D. J., McMillan C. T., Harms M. B., Cairns N. J., Wood E. M. et al. (2015). Hypermethylation of repeat expanded C9orf72 is a clinical and molecular disease modifier. *Acta Neuropathol.* 129, 39-52. 10.1007/s00401-014-1365-0 25388784 PMC4282973

[DMM028613C127] Russell A. P., Wada S., Vergani L., Hock M. B., Lamon S., Léger B., Ushida T., Cartoni R., Wadley G. D., Hespel P. et al. (2013). Disruption of skeletal muscle mitochondrial network genes and miRNAs in amyotrophic lateral sclerosis. *Neurobiol. Dis.* 49, 107-117. 10.1016/j.nbd.2012.08.015 22975021

[DMM028613C128] Sareen D., O'Rourke J. G., Meera P., Muhammad A. K. M. G., Grant S., Simpkinson M., Bell S., Carmona S., Ornelas L., Sahabian A. et al. (2013). Targeting RNA foci in iPSC-derived motor neurons from ALS patients with a C9ORF72 repeat expansion. *Sci. Transl. Med.* 5, 208ra149 10.1126/scitranslmed.3007529 PMC4090945 24154603

[DMM028613C129] Scekic-Zahirovic J., Sendscheid O., El Oussini H., Jambeau M., Sun Y., Mersmann S., Wagner M., Dieterlé S., Sinniger J., Dirrig-Grosch S. et al. (2016). Toxic gain of function from mutant FUS protein is crucial to trigger cell autonomous motor neuron loss. *EMBO J.* 35, 1077-1097. 10.15252/embj.201592559 26951610 PMC4868956

[DMM028613C130] Schaffer A. E., Eggens V. R. C., Caglayan A. O., Reuter M. S., Scott E., Coufal N. G., Silhavy J. L., Xue Y., Kayserili H., Yasuno K. et al. (2014). CLP1 founder mutation links tRNA splicing and maturation to cerebellar development and neurodegeneration. *Cell* 157, 651-663. 10.1016/j.cell.2014.03.049 24766810 PMC4128918

[DMM028613C131] Schonrock N., Humphreys D. T., Preiss T. and Götz J. (2012). Target gene repression mediated by miRNAs miR-181c and miR-9 both of which are down-regulated by amyloid-β. *J. Mol. Neurosci.* 46, 324-335. 10.1007/s12031-011-9587-2 21720722

[DMM028613C132] Schwarz D., Hutvágner G., Du T., Xu Z., Aronin N. and Zamore P. D. (2003). Asymmetry in the assembly of the RNAi enzyme complex. *Cell* 115, 199-208. 10.1016/S0092-8674(03)00759-1 14567917

[DMM028613C133] Sellier C., Rau F., Liu Y., Tassone F., Hukema R. K., Gattoni R., Schneider A., Richard S., Willemsen R., Elliott D. J. et al. (2010). Sam68 sequestration and partial loss of function are associated with splicing alterations in FXTAS patients. *EMBO J.* 29, 1248-1261. 10.1038/emboj.2010.21 20186122 PMC2857464

[DMM028613C134] Sellier C., Freyermuth F., Tabet R., Tran T., He F., Ruffenach F., Alunni V., Moine H., Thibault C., Page A. et al. (2013). Sequestration of DROSHA and DGCR8 by expanded CGG RNA repeats alters microRNA processing in fragile X-associated tremor/ataxia syndrome. *Cell Rep.* 3, 869-880. 10.1016/j.celrep.2013.02.004 23478018 PMC3639429

[DMM028613C135] Sephton C. F., Cenik C., Kucukural A., Dammer E. B., Cenik B., Han Y., Dewey C. M., Roth F. P., Herz J., Peng J. et al. (2011). Identification of neuronal RNA targets of TDP-43-containing ribonucleoprotein complexes. *J. Biol. Chem.* 286, 1204-1215. 10.1074/jbc.M110.190884 21051541 PMC3020728

[DMM028613C136] Sharma A., Lyashchenko A., Lu L., Nasrabady S. E., Elmaleh M., Mendelsohn M., Nemes A., Tapia J. C., Mentis G. Z. and Shneider N. A. (2016). ALS-associated mutant FUS induces selective motor neuron degeneration through toxic gain of function. *Nat. Commun.* 7, 10465 10.1038/ncomms10465 26842965 PMC4742863

[DMM028613C137] Shiga A., Ishihara T., Miyashita A., Kuwabara M., Kato T., Watanabe N., Yamahira A., Kondo C., Yokoseki A., Takahashi M. et al. (2012). Alteration of POLDIP3 splicing associated with loss of function of TDP-43 in tissues affected with ALS. *PLoS ONE* 7, e43120 10.1371/journal.pone.0043120 22900096 PMC3416794

[DMM028613C138] Shioya M., Obayashi S., Tabunoki H., Arima K., Saito Y., Ishida T. and Satoh J. (2010). Aberrant microRNA expression in the brains of neurodegenerative diseases: miR-29a decreased in Alzheimer disease brains targets neurone navigator 3. *Neuropathol. Appl. Neurobiol.* 36, 320-330. 10.1111/j.1365-2990.2010.01076.x 20202123

[DMM028613C139] Smith J., Calidas D., Schmidt H., Lu T., Rasoloson D. and Seydoux G. (2016). Spatial patterning of P granules by RNA-induced phase separation of the intrinsically-disordered protein MEG-3. *Elife* 5, e21337 10.7554/elife.21337 27914198 PMC5262379

[DMM028613C140] Sofola O. A., Jin P., Qin Y., Duan R., Liu H., de.Haro M., Nelson D. L. and Botas J. (2007). RNA-binding proteins hnRNP A2/B1 and CUGBP1 suppress fragile X CGG premutation repeat-induced neurodegeneration in a Drosophila model of FXTAS. *Neuron* 55, 565-571. 10.1016/j.neuron.2007.07.021 17698010 PMC2215388

[DMM028613C141] Sreedharan J., Blair I. P., Tripathi V. B., Hu X., Vance C., Rogelj B., Ackerley S., Durnall J. C., Williams K. L., Buratti E. et al. (2008). TDP-43 mutations in familial and sporadic amyotrophic lateral sclerosis. *Science* 319, 1668-1672. 10.1126/science.1154584 18309045 PMC7116650

[DMM028613C142] Strong M. J., Volkening K., Hammond R., Yang W., Strong W., Leystra-Lantz C. and Shoesmith C. (2007). TDP43 is a human low molecular weight neurofilament (hNFL) mRNA-binding protein. *Mol. Cell. Neurosci.* 35, 320-327. 10.1016/j.mcn.2007.03.007 17481916

[DMM028613C143] Su Z., Zhang Y., Gendron T. F., Bauer P. O., Chew J., Yang W.-Y., Fostvedt E., Jansen-West K., Belzil V. V., Desaro P. et al. (2014). Discovery of a biomarker and lead small molecules to target r(GGGGCC)-associated defects in c9FTD/ALS. *Neuron* 83, 1043-1050. 10.1016/j.neuron.2014.07.041 25132468 PMC4232217

[DMM028613C144] Sullenger B. A. and Nair S. (2016). From the RNA world to the clinic. *Science* 352, 1417-1420. 10.1126/science.aad8709 27313039 PMC6035743

[DMM028613C145] Sun Z., Diaz Z., Fang X., Hart M. P., Chesi A., Shorter J. and Gitler A. D. (2011). Molecular determinants and genetic modifiers of aggregation and toxicity for the ALS disease protein FUS/TLS. *PLoS Biol.* 9, e1000614 10.1371/journal.pbio.1000614 21541367 PMC3082519

[DMM028613C146] Sunwoo J.-S., Lee S.-T., Im W., Lee M., Byun J.-I., Jung K.-H., Park K.-I., Jung K.-Y., Lee S., Chu K. et al. (2016). Altered expression of the long noncoding RNA NEAT1 in huntington's disease. *Mol. Neurobiol.* 54, 1577-1586. 10.1007/s12035-016-9928-9 27221610

[DMM028613C147] Tan J. Y., Vance K. W., Varela M. A., Sirey T., Watson L. M., Curtis H. J., Marinello M., Alves S., Steinkraus B. R., Cooper S. et al. (2014). Cross-talking noncoding RNAs contribute to cell-specific neurodegeneration in SCA7. *Nat. Struct. Mol. Biol.* 21, 955-961. 10.1038/nsmb.2902 25306109 PMC4255225

[DMM028613C148] Taneja K. L., McCurrach M., Schalling M., Housman D. and Singer R. H. (1995). Foci of trinucleotide repeat transcripts in nuclei of myotonic dystrophy cells and tissues. *J. Cell Biol.* 128, 995-1002. 10.1083/jcb.128.6.995 7896884 PMC2120416

[DMM028613C149] Thum T. (2012). MicroRNA therapeutics in cardiovascular medicine. *EMBO Mol. Med.* 4, 3-14. 10.1002/emmm.201100191 22162462 PMC3376835

[DMM028613C150] Toivonen J. M., Manzano R., Oliván S., Zaragoza P., García-Redondo A. and Osta R. (2014). MicroRNA-206: a potential circulating biomarker candidate for amyotrophic lateral sclerosis. *PLoS ONE* 9, e89065 10.1371/journal.pone.0089065 24586506 PMC3930686

[DMM028613C151] Tollervey J. R., Curk T., Rogelj B., Briese M., Cereda M., Kayikci M., König J., Hortobágyi T., Nishimura A. L., Zupunski V. et al. (2011). Characterizing the RNA targets and position-dependent splicing regulation by TDP-43. *Nat. Neurosci.* 14, 452-458. 10.1038/nn.2778 21358640 PMC3108889

[DMM028613C152] Tripathi V., Ellis J. D., Shen Z., Song D. Y., Pan Q., Watt A. T., Freier S. M., Bennett P. A., Sharma A., Bubulya P. A. et al. (2010). The nuclear-retained noncoding RNA MALAT1 regulates alternative splicing by modulating SR splicing factor phosphorylation. *Mol. Cell* 39, 925-938. 10.1016/j.molcel.2010.08.011 20797886 PMC4158944

[DMM028613C153] Trotta C., Paushkin S. V., Patel M., Li H. and Peltz S. W. (2006). Cleavage of pre-tRNAs by the splicing endonuclease requires a composite active site. *Nature* 441, 375-377. 10.1038/nature04741 16710424

[DMM028613C154] Tsai K.-J., Yang C.-H., Fang Y.-H., Cho K.-H., Chien W.-L., Wang W.-T., Wu T.-W., Lin C.-P., Fu W.-M. and Shen C.-K. (2010). Elevated expression of TDP-43 in the forebrain of mice is sufficient to cause neurological and pathological phenotypes mimicking FTLD-U. *J. Exp. Med.* 207, 1661-1673. 10.1084/jem.20092164 20660618 PMC2916125

[DMM028613C155] Van Deerlin V. M., Leverenz J. B., Bekris L. M., Bird T. D., Yuan W., Elman L. B., Clay D., Wood E. M., Chen-Plotkin A. S., Martinez-Lage M. et al. (2008). TARDBP mutations in amyotrophic lateral sclerosis with TDP-43 neuropathology: a genetic and histopathological analysis. *Lancet. Neurol.* 7, 409-416. 10.1016/S1474-4422(08)70071-1 18396105 PMC3546119

[DMM028613C156] Vance C., Rogelj B., Hortobágyi T., De Vos K. J., Nishimura A. L., Sreedharan J., Hu X., Smith B., Ruddy D., Wright P. et al. (2009). Mutations in FUS, an RNA processing protein, cause familial amyotrophic lateral sclerosis type 6. *Science* 323, 1208-1211. 10.1126/science.1165942 19251628 PMC4516382

[DMM028613C157] Vassar R., Bennett B. D., Babu-Khan S., Kahn S., Mendiaz E. D., Denis P., Teplow D. B., Ross S., Amarante P., Loeloff R. et al. (1999). Beta-secretase cleavage of Alzheimer's amyloid precursor protein by the transmembrane aspartic protease BACE. *Science* 286, 735-741. 10.1126/science.286.5440.735 10531052

[DMM028613C158] Voigt A., Herholz D., Fiesel F. C., Kaur K., Müller D., Karsten P., Weber S. S., Kahle P. J., Marquardt T. and Schulz J. B. (2010). TDP-43-mediated neuron loss in vivo requires RNA-binding activity. *PLoS ONE* 5, e12247 10.1371/journal.pone.0012247 20806063 PMC2923622

[DMM028613C159] Wang W.-X., Rajeev B. W., Stromberg A. J., Ren N., Tang G., Huang Q., Rigoutsos I. and Nelson P. T. (2008). The expression of microRNA miR-107 decreases early in Alzheimer's disease and may accelerate disease progression through regulation of beta-site amyloid precursor protein-cleaving enzyme 1. *J. Neurosci.* 28, 1213-1223. 10.1523/JNEUROSCI.5065-07.2008 18234899 PMC2837363

[DMM028613C160] Wang W.-X., Huang Q., Hu Y., Stromberg A. J. and Nelson P. T. (2011). Patterns of microRNA expression in normal and early Alzheimer's disease human temporal cortex: white matter versus gray matter. *Acta Neuropathol.* 121, 193-205. 10.1007/s00401-010-0756-0 20936480 PMC3073518

[DMM028613C161] Wang E. T., Cody N. A. L., Jog S., Biancolella M., Wang T. T., Treacy D. J., Luo S., Schroth G. P., Housman D. E., Reddy S. et al. (2012a). Transcriptome-wide regulation of pre-mRNA splicing and mRNA localization by muscleblind proteins. *Cell* 150, 710-724. 10.1016/j.cell.2012.06.041 22901804 PMC3428802

[DMM028613C162] Wang I.-F., Guo B.-S., Liu Y.-C., Wu C.-C., Yang C.-H., Tsai K.-J. and Shen C.-K. J. (2012b). Autophagy activators rescue and alleviate pathogenesis of a mouse model with proteinopathies of the TAR DNA-binding protein 43. *Proc. Natl. Acad. Sci. USA* 109, 15024-15029. 10.1073/pnas.1206362109 22932872 PMC3443184

[DMM028613C163] Watts G. D., Wymer J., Kovach M. J., Mehta S. G., Mumm S., Darvish D., Pestronk A., Whyte M. P. and Kimonis V. E. (2004). Inclusion body myopathy associated with Paget disease of bone and frontotemporal dementia is caused by mutant valosin-containing protein. *Nat. Genet.* 36, 377-381. 10.1038/ng1332 15034582

[DMM028613C164] Wheeler T. M., Leger A. J., Pandey S. K., MacLeod A. R., Nakamori M., Cheng S. H., Wentworth B. M., Bennett C. F. and Thornton C. A. (2012). Targeting nuclear RNA for in vivo correction of myotonic dystrophy. *Nature* 488, 111-115. 10.1038/nature11362 22859208 PMC4221572

[DMM028613C165] Wils H., Kleinberger G., Janssens J., Pereson S., Joris G., Cuijt I., Smits V., Ceuterick-de Groote C., Broeckhoven C. and Kumar-Singh S. (2010). TDP-43 transgenic mice develop spastic paralysis and neuronal inclusions characteristic of ALS and frontotemporal lobar degeneration. *Proc. Natl. Acad. Sci. USA* 107, 3858-3863. 10.1073/pnas.0912417107 20133711 PMC2840518

[DMM028613C166] Wu W., Sun M., Zou G.-M. and Chen J. (2007a). MicroRNA and cancer: current status and prospective. *Int. J. Cancer* 120, 953-960. 10.1002/ijc.22454 17163415

[DMM028613C167] Wu D., Yu W., Kishikawa H., Folkerth R. D., Iafrate J., Shen Y., Xin W., Sims K. and Hu G. (2007b). Angiogenin loss-of-function mutations in amyotrophic lateral sclerosis. *Ann. Neurol.* 62, 609-617. 10.1002/ana.21221 17886298 PMC2776820

[DMM028613C168] Xiang S., Kato M., Wu L. C., Lin Y., Ding M., Zhang Y., Yu Y. and McKnight S. L. (2015). The LC domain of hnRNPA2 adopts similar conformations in hydrogel polymers, liquid-like droplets, and nuclei. *Cell* 163, 829-839. 10.1016/j.cell.2015.10.040 26544936 PMC4879888

[DMM028613C169] Xie W., Nangle L. A., Zhang W., Schimmel P. and Yang X.-L. (2007). Long-range structural effects of a Charcot-Marie-Tooth disease-causing mutation in human glycyl-tRNA synthetase. *Proc. Natl. Acad. Sci. USA* 104, 9976-9981. 10.1073/pnas.0703908104 17545306 PMC1891255

[DMM028613C170] Xu Y.-F., Gendron T., Zhang Y.-J., Lin W.-L., D'Alton S., Sheng H., Casey M. C., Tong J., Knight J., Yu X. et al. (2010). Wild-type human TDP-43 expression causes TDP-43 phosphorylation, mitochondrial aggregation, motor deficits, and early mortality in transgenic mice. *J. Neurosci.* 30, 10851-10859. 10.1523/JNEUROSCI.1630-10.2010 20702714 PMC3056148

[DMM028613C177] Yang M., Liang C., Swaminathan K., Herrlinger S., Lai F., Shiekhattar R., and Chen J.-F. (2016). A C9ORF72/SMCR8-containing complex regulates ULK1 and plays a dual role in autophagy. *Science Advances* 2, e1601167 10.1126/sciadv.1601167 27617292 PMC5010369

[DMM028613C171] Yamasaki S., Ivanov P., Hu G. F. and Anderson P. (2009). Angiogenin cleaves tRNA and promotes stress-induced translational repression. *J. Cell. Biol.* 185, 35-42. 10.1083/jcb.200811106 19332886 PMC2700517

[DMM028613C172] Zhou H., Huang C., Chen H., Wang D., Landel C. P., Xia P., Bowser R., Liu Y.-J. and Xia X. G. (2010). Transgenic rat model of neurodegeneration caused by mutation in the TDP gene. *PLoS Genet.* 6, e1000887 10.1371/journal.pgen.1000887 20361056 PMC2845661

[DMM028613C173] Zovoilis A., Agbemenyah H. Y., Agis-Balboa R. C., Stilling R. M., Edbauer D., Rao P., Farinelli L., Delalle I., Schmitt A., Falkai P. et al. (2011). microRNA-34c is a novel target to treat dementias. *EMBO J.* 30, 4299-4308. 10.1038/emboj.2011.327 21946562 PMC3199394

[DMM028613C174] Zu T., Gibbens B., Doty N. S., Gomes-Pereira M., Huguet A., Stone M. D., Margolis J., Peterson M., Markowski T. W., Ingram M. A. et al. (2011). Non-ATG-initiated translation directed by microsatellite expansions. *Proc. Natl. Acad. Sci. USA* 108, 260-265. 10.1073/pnas.1013343108 21173221 PMC3017129

[DMM028613C175] Zu T., Liu Y., Bañez-Coronel M., Reid T., Pletnikova O., Lewis J., Miller T. M., Harms M. B., Falchook A. E., Subramony S. H. et al. (2013). RAN proteins and RNA foci from antisense transcripts in C9ORF72 ALS and frontotemporal dementia. *Proc. Natl. Acad. Sci. USA* 110, E4968-E4977. 10.1073/pnas.1315438110 24248382 PMC3870665

